# Recent antitumor therapy does not increase Omicron COVID-19 severity in cancer patients: a two-center retrospective study in China

**DOI:** 10.3389/fonc.2023.1284255

**Published:** 2023-11-21

**Authors:** Ying Liu, Wenyao Zhu, Zhiwu Wang, Jiarui Yu, Liang Dong, Chunyang Li, Wei Wang, Fuhui Zhang, Shuanghui Ding, Lu Sun, Zhao Yang, Chao Zhang, Fang Qian

**Affiliations:** ^1^ Department of Infectious Disease, The First Affiliated Hospital of Soochow University, Suzhou, China; ^2^ Department of Radiotherapy, Yantai Yuhuangding Hospital, Yantai, China; ^3^ Department of Oncology, Tangshan People’s Hospital, Tangshan, China

**Keywords:** coronavirus disease 2019 (COVID-19), omicron, severe acute respiratory syndrome coronavirus-2 (SARS-CoV-2), clinical features, anticancer therapy, chemotherapy

## Abstract

**Background:**

The impact of anticancer therapy and related clinical factors on the severity of COVID-19 in cancer patients during the Omicron pandemic has not been established. The recent outbreak in China caused predominantly by the BA.5.2 and BF.7 strains of Omicron provided us with the opportunity to observe objectively the impact of this strain in oncology patients. We initiated this two–center retrospective study in China to determine the impact of anti-cancer treatment, other clinical factors, and cancer characteristics on COVID-19 severity in cancer patients infected with coronavirus during the SARS-CoV-2 Omicron variant pandemic in China.

**Methods:**

We retrospectively included 554 cancer patients infected with COVID-19 from two medical centers. Data on their anticancer treatment prior to COVID-19 infection and general clinical characteristics (sex, age, past medical history, etc.) were collected. Univariate statistical analysis was performed to identify the factors associated with the severity of COVID-19.

**Results:**

Among 554 cancer patients infected with COVID-19, there were 15 (2.7%) severe/critical cases, 86 (15.5%) cases with medium severity, and 453 (81.8%) cases with mild severity. Univariate analysis revealed that advanced age, male sex, worse ECOG score, unvaccinated status, and previous liver, kidney, and brain diseases were associated with more severe COVID-19. However, recent antitumor therapy, including cytotoxic chemotherapy within two weeks did not have a significant correlation with the severity of COVID-19 caused by the Omicron variant.

**Conclusion:**

The severity of COVID-19 caused by the Omicron variant is not exacerbated by recent anticancer therapy in cancer patients. Therefore, anticancer therapy should not be discontinued in such cases, especially those with mild severity.

## Graphical Abstract

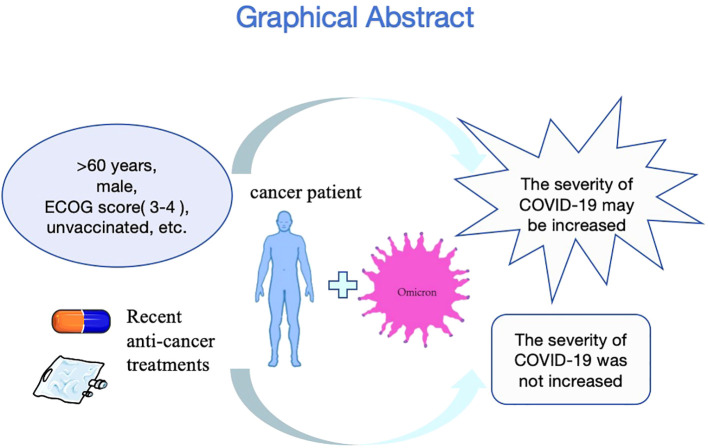

## Introduction

Following the emergence of severe acute respiratory syndrome coronavirus 2 (SARS-CoV-2) at the end of 2019, the COVID-19 pandemic posed a serious threat and a huge challenge to global public health. Moreover, the evolution and mutations of SARS-CoV-2 since late 2020 have affected the characteristics of the virus, including its transmissibility and pathogenicity. A series of new SARS-CoV-2 variants pose different levels of threat to COVID-19 morbidity, mortality, and socioeconomic burden (1). Among them, the Omicron variant is the most variable variant of SARS-CoV-2 (2), which has caused global panic and concern because of its high infectivity and immune evasion ability since it was reported by the World Health Organization in 2021 (3, 4). Currently, Omicron has rapidly replaced Delta as the leading variant, and the prevalence of SARS-CoV-2 Omicron variant remains a serious global health concern.

Previous laboratory studies have shown that Omicron is more likely to infect the upper respiratory tract than the lung parenchyma, making the virus less pathogenic but potentially facilitating its higher transmissibility (5–8). This speculation has also been confirmed in clinical studies (2). Indeed, many investigations have found that the pathogenicity of Omicron variant is significantly lower than that of the original strain, and the probability of severe disease and death is significantly lower than that of caused by the Beta or Delta variant (2, 3). However, it is noteworthy that the impact of Omicron is not diminished by its reduced pathogenicity, and the health care system is still under great pressure due to the high transmissibility of the Omicron variant strains (3, 7). Cancer patients have been at high risk for serious illness and death from COVID-19 infection. The detrimental effect of Omicron infection on cancer patients with advanced age, advanced tumor stages, and increased comorbidities was reported earlier (9). Protecting cancer patients from the adverse effects of SARS-CoV-2 infection has been a top priority for oncologists since early 2020. The safety of cancer therapy also deserves further attention in the context of the increased risk of Omicron transmission. The cancer patient population is heterogeneous, and a better understanding of the specific risk factors associated with adverse COVID-19 outcomes in cancer patients may facilitate clinical management.

Previous studies have analyzed the adverse effects of cancer treatment on COVID-19 outcomes (10–13). However, most of these data have been obtained in studies focused on infection with the original and early mutant strains of the new coronavirus, and their findings have been conflicting. The recent outbreak in China caused predominantly by the BA.5.2 and BF.7 strains of Omicron provided us with the opportunity to observe objectively the impact of this strain in oncology patients. Therefore, we initiated this two-center retrospective study in China to establish the impact of anti-cancer treatment, clinical factors, and cancer characteristics on COVID-19 severity in cancer patients in China infected with the Omicron variant. Our findings would help oncologists to more accurately assess the condition of patients infected with the new coronavirus, implement individualized treatment, and to use new strategies to maintain antitumor therapy for cancer patients during epidemics of the COVID-19 Omicron variant.

## Methods

In this retrospective observational study, we analyzed the data of a total number of 554 cancer patients who were treated in the Chemoradiotherapy Department of Tangshan People’s Hospital from September to December 2022 and the Radiotherapy Department of Yantai Yuhuangding Hospital in December 2022, all of whom underwent nasal or throat sampling and were confirmed positive for SARS-CoV-2 by RT-PCR or had undergone antigen testing. All patients were followed up by the responsible physicians, the COVID-19 symptoms and development were recorded, and its severity was assessed. Patients were classified into “light, medium, heavy, and critical” according to the clinical staging of the Chinese Clinical Protocol for the Treatment of Novel Coronavirus Infection (Trial Version 10), detailed in Supplementary File 1*. We collected general clinical information of the patients, including age, sex, history of other diseases (cardiovascular disease, hypertension, diabetes mellitus, and other diseases), ECOG (Eastern Cooperative Oncology Group) score, and vaccination information. We also collected information concerning tumors and their treatment, including type of the tumor, tumor stage at the time of COVID-19 diagnosis, and the timing and type of recent anticancer treatment within three months before COVID-19 diagnosis. The timing of anticancer treatment was categorized as follows: within 2 weeks, within 2–4 weeks, 1–3 months, and >3 months prior to COVID-19 diagnosis. Anti-cancer modalities were defined as cytotoxic chemotherapy, immunotherapy, targeted therapy, radiotherapy, and combination therapy. The final follow-up date was January 10, 2023.

This study was approved by ethics committee of Tangshan People’s Hospital and all methods were carried out in accordance with the ethical rules of the Helsinki Declaration and Good Clinical Practice. Written informed consent was waived by the ethics committee of Tangshan People’s Hospital because this was a retrospective, non-invasive and observational study.

### Statistical methods

Normally distributed variables were expressed as $\bar{\text{x}}$ ± s, and *t*-test was used for comparison between groups. Non-normally distributed variables were expressed as M (interquartile range [IQR]), and Wilcoxon rank-sum test was applied for comparison between groups. Statistical analyses were performed using SPSS19.0. All statistical tests were two-sided, and *P* < 0.05 was considered to indicate a statistically significant difference.

## Results

In this study, we initially collected the data of 761 cancer patients (632 in Tangshan People’s Hospital and 129 in Yantai Yuhuangding Hospital), excluding patients who were lost to follow-up and not infected with COVID-19. Finally, 554 cancer patients with SARS-CoV-2 infection were enrolled, including 453 in Tangshan People’s Hospital and 101 in Yantai Yuhuangding Hospital. The statistical data of their symptoms, disease durations, and clinical staging are presented in **Table 1**. There were 453 mild cases (81.8%), 86 moderate cases (15.5%), and 15 severe and critical cases (2.7%). Of the severe and critical patients, 13 (2.3%) died. A total number of 138 (24.9%) patients in this investigation cohort were symptomatic but not febrile. A number of 320 (57.8%) had low/moderate fever, whereas 96 (17.3%) had high fever; 59 (10.6%) had dyspnea or wheezing. Imaging studies were performed in 167 (30.1%) of 554 patients and radiographic changes were found in 59 (10.6%) of the patients. The majority of the patients took antipyretic and analgesic drugs and proprietary Chinese medicines outside the hospital, and 103 (18.6%) of them were treated with antibiotics; 25 (4.5%) patients chose hospitalization due to more severe symptoms, but none of them was treated with antiviral drugs such as nematovir tablets/ritonavir (PAXLOVID) in the early stage of infection.

**Table 1:** COVID–19 symptoms, course, and clinical staging of cancer patients.

Variables	Quantity
Symptoms	
No fever	138 (24.9)
Low/Medium fever	320 (57.8)
High fever	96 (17.3)
Cough/dry cough	367 (66.2)
Sputum present	252 (45.5)
Fatigue/weakness	444 (80.1)
Difficulty breathing/wheezing	60 (10.8)
Chest tightness	85 (15.3)
Sore throat	221 (39.9)
Diarrhea	69 (12.4)
Nausea/vomiting	37 (6.7)
Stuffy and runny nose	136 (24.5)
Chilliness/chill fear	205 (37.0)
Headache/Myalgia	232 (41.9)
Altered sense of smell	89 (16.1)
Altered sense of taste	182 (32.9)
Course of disease	
Imaging was performed	167 (30.1)
With imaging changes	59 (10.6)
Inpatient treatment	25 (4.5)
Oxygen therapy	21 (3.8)
Antibiotic treatment	103 (18.6)
Treatment with glucocorticoids	14 (2.5)
Clinical staging of COVID-19	
Mild	453 (81.8)
Moderate	86 (15.5)
Severe/Critical	15 (2.7)
Disease regression	
Progression	541 (97.7)
Death	13 (2.3)

Data in () are percentages (%).

The demographics data of the patients infected with COVID–19 are presented in **Table 2**. There were 321 (57.9%) male patients and 233 (42.1%) female patients, with a median age of 60 years (IQR 55–67). Six patients had febrile neutropenia during the course of their infection with COVID–19.

**Table 2:** Clinical characteristics of cancer patients with different severity of COVID-19 infection.

Clinical variables	Total number	COVID-19 Severity		
	554	Mild	Moderate	Severe/Critical
**Sex**	554	453	86	15
Male	321 (57.9)	251 (55.4)	57 (66.3)	13 (86.7)
Female	233 (42.1)	202 (44.6)	29 (33.7)	2 (13.3)
**Age median (IQR)**	60 (55–67)	60 (54–67)	62.5 (57.75–69.25)	70 (64–74)
**≤60 years**	284 (51.3)	246 (54.3)	36 (41.9)	2 (13.3)
**>60 years**	270 (48.7)	207 (45.7)	50 (58.1)	13 (86.7)
**ECOG score**	554	453	86	15
0–2	531 (95.8)	443 (97.8)	77 (89.5)	11 (73.3)
3–4	23 (4.2)	10 (2.2)	9 (10.5)	4 (26.7)
**Comorbidities**	554	453	86	15
Cardiovascular disease	39 (7.0)	26 (5.7)	10 (11.6)	3 (20.0)
Hypertension	141 (25.5)	116 (25.6)	21 (24.4)	4 (26.7)
Diabetes	63 (11.4)	50 (11)	12 (14.0)	1 (6.7)
COPD/emphysema	18 (3.2)	15 (3.3)	2 (2.3)	1 (6.7)
Others (liver, kidney, brain, etc.)	114 (20.6)	88 (19.4)	19 (22.1)	7 (46.7)
Cardiovascular disease & hypertension	23 (4.2)	17 (3.8)	4 (4.7)	2 (13.3)
**Tumor type**	554	453	86	15
Lung cancer	174 (31.4)	126 (27.8)	44 (51.2)	4 (26.7)
Others	380 (68.6)	327 (72.2)	42 (48.8)	11 (73.3)
**Tumor stage (whether stage IV)**	554	453	86	15
Yes	264 (47.7)	205 (45.3)	50 (58.1)	9 (60.0)
No	290 (52.3)	248 (54.7)	36 (41.9)	6 (40.0)
**Time of cancer diagnosis**	554	453	86	15
≤1 year	351 (63.4)	293 (64.7)	48 (55.8)	10 (66.6)
1–5 years	180 (32.5)	141 (31.1)	35 (40.7)	4 (26.7)
>5 years	23 (4.2)	19 (4.2)	3 (3.5)	1 (6.7)
**Nature of treatment before infection**	554	453	86	15
Radical treatment	78 (14.1)	68 (15.0)	8 (9.3)	2 (13.3)
Adjuvant	94 (17.0)	83 (18.3)	9 (10.5)	2 (13.3)
Palliative	276 (49.8)	219 (48.3)	47 (54.4)	10 (66.7)
Untreated within 3 months	106 (19.1)	85 (18.8)	20 (23.3)	1 (6.7)
Treatment modality (Within 3 months^a^)				
Chemotherapy	333 (60.1)	272 (60.0)	51 (59.3)	10 (66.7)
Targeted therapy	136 (24.5)	105 (23.2)	27 (31.4)	4 (26.7)
Immunotherapy	117 (21.1)	89 (19.6)	22 (25.6)	6 (40.0)
Chemotherapy&targeted therapy	77 (13.9)	58 (12.8)	16 (18.6)	3 (20.0)
Chemotherapy&Immunotherapy	91 (16.4)	71 (15.7)	18 (20.9)	2 (13.3)
Chest radiation therapy	33 (6.0)	32 (21.0)	0 (0)	1 (6.7)
**Time to last treatment^a^**	554	453	86	15
≤1 month	257 (46.4)	209 (46.1)	40 (46.5)	8 (53.3)
1–3 months	191 (34.5)	158 (34.9)	26 (30.2)	6 (40.0)
>3 months	106 (19.1)	85 (18.8)	20 (23.3)	1 (6.7)
**Time to last cytotoxic chemotherapy^a^**	333	272	51	10
≤2 weeks	109 (32.7)	87 (32.0)	18 (35.3)	4 (40.0)
2–4 weeks	77 (23.1)	66 (24.3)	9 (17.6)	2 (20.0)
1–3 months	147 (44.1)	119 (43.8)	24 (47.1)	4 (40.0)
**Time to last immunotherapy**	117	89	22	6
≤2 weeks	36 (30.8)	29 (32.6)	6 (27.3)	2 (33.3)
2–4 weeks	27 (23.1)	23 (25.8)	2 (9.1)	2 (33.3)
1–3 months	54 (46.2)	37 (41.6)	14 (63.6)	2 (33.3)
**Vaccination status**	554	453	86	15
Not vaccinated	232 (41.9)	180 (39.7)	40 (46.5)	12 (80.0)
Vaccinated	322 (58.1)	273 (60.3)	46 (53.5)	3 (20.0)
**Time of the last vaccination**	322	273	46	3
≤12 months	128 (39.8)	109 (39.9)	17 (37.0)	2 (66.7)
>12 months	194 (60.2)	164 (60.1)	29 (63.0)	1 (33.3)
Presence of febrile neutropenia *	6 (1.1)	3 (0.7)	2 (2.3)	1 (6.7)

Data in () are percentages (%); **^a^** before COVID-19 diagnosis; *one week prior to COVID-19 infection to recovery from infection.

We assessed the severity of the disease based on the most severe disease status reported by the patients and compared the data of severely/critically ill patients with those of mild/medium patients, analyzing their clinical characteristics and received recent anticancer treatment (**Table 3**). The data on the correlation between clinical characteristics and anticancer treatment of cancer patients with COVID-19 severity (severe/critical vs. mild/medium type) as shown in **Table 3**. In our univariate analysis, we found that patients’ sex, age, ECOG score, and other types of comorbidities, as well as the vaccination status were statistically different between the two groups of patients. The patients in the severe/critical category were significantly older (median 70 years (IQR 64–74) vs 60 years (IQR 54–67); *P* = 0.003), and there were more male (13 of 15 patients than female (2 [13.3%]); *P* = 0.02) in the severe/critical category [86.7%]). The patients in the severe/critical category had worse ECOG scores (3 to 4 vs 0 to 2 ratio OR 9.95 [95% CI 2.90–34.14]; *P* < 0.001), and the number of unvaccinated patients in the severe/critical type group was larger than in that of the vaccinated patients (12 vs 3; *P* = 0.02).

**Table 3:** Univariate analysis of clinical characteristics and anticancer treatment of cancer patients with COVID-19 severity (severe/critical vs. mild/medium type).

Clinical variables	*OR* (95% CI)	*P*
**Sex (male vs female)**	4.88 (1.09–21.81)	0.02
**Age (>60 years vs ≤60 years)**	7.13 (1.59–31.91)	0.003
**ECOG score (3–4 vs 0–2)**	9.95 (2.90–34.14)	<0.001
Comorbidities (Yes vs No)		
Cardiovascular disease	3.49 (0.94–12.94)	0.14
Hypertension	1.07 (0.33–3.41)	1.0
Diabetes mellitus	0.55 (0.07–4.25)	0.87
COPD/emphysema	2.19 (0.27–17.65)	0.40
Others (liver, kidney, brain, etc.)	3.53 (1.25–9.95)	0.03
Cardiovascular disease & hypertension	3.62 (0.77–17.01)	0.27
**Tumor type (lung cancer vs others)**	0.79 (0.25–2.52)	0.91
**Whether the tumor stage is IV (Yes vs No)**	1.67 (0.59–4.76)	0.33
**Time of cancer diagnosis (≤1 year vs > 1 year)**	1.16 (0.39–3.45)	0.79
**Nature of treatment (palliative vs. radical vs. adjuvant)**	——	0.72
Treatment modality (Within 3 months^a^)		
Chemotherapy **(Yes vs No)**	1.34 (0.45–3.97)	0.60
Targeted therapy **(Yes vs No)**	1.12 (0.35–3.58)	1.0
Immunotherapy **(Yes vs No)**	2.57 (0.90–7.37)	0.14
Chemotherapy&targeted therapy **(Yes vs No)**	1.57 (0.43–5.70)	0.75
Chemotherapy&Immunotherapy **(Yes vs No)**	0.78 (0.17–3.51)	1.0
Chest radiation therapy **(Yes vs No)**	1.13 (0.14–8.88)	0.61
Time to last treatment^a^		
≤2 weeks vs 2–4 weeks	0.47 (0.12–1.94)	0.50
≤2 weeks vs1–3 months	0.73 (0.20–2.63)	0.87
≤2 weeks vs >3 months	2.49 (0.27–22.54)	0.71
**Time to last cytotoxic chemotherapy^a^ (**≤2 weeks vs 2–4 weeks vs 1–3 months)		0.89
≤2 weeks vs 2–4 weeks	1.43 (0.26–8.00)	1.0
≤2 weeks vs 1–3 months	1.36 (0.33–5.57)	0.95
**Time to last cytotoxic chemotherapy^a^ (**≤2 weeks vs 2–4 weeks vs1–3 months)		0.77
≤2 weeks vs 2–4 weeks	0.74 (0.10–5.58)	1.0
≤2 weeks vs 1–3 months	1.53 (0.21–11.38)	1.0
**Vaccination (vaccinated vs. not vaccinated)**	0.17 (0.05–0.62)	0.02
**Time to last vaccination (≤1 year vs >1 year)**	3.06 (0.28–34.14)	0.72

**^a^** before COVID-19 diagnosis.

We analyzed the anticancer treatment of cancer patients with COVID-19 and established the severity of COVID-19 was not associated with the treatment patterns in the last three months. The severity of COVID-19 had not increased even after the patient had received chemotherapy within two weeks before infection.

## Discussion

COVID-19 poses a significant risk to cancer patients. The novel SARS-CoV-2 Omicron variant has become the most prevalent worldwide. Understanding the factors associated with a high risk of severe COVID-19 disease is critical to ensuring better protection of cancer patients, and can provide valuable guidance in taking clinical treatment decisions. Although the pathogenicity of the Omicron variant has been greatly reduced relative to the original strain, the overall risk to cancer patients remains a concern due to its highly infectious nature. Notably, because of the consistently low prevalence of coexisting cancer and COVID-19, only a small number of patients with both diseases has been generally established. In particular, many unknown clinical symptoms have been observed, and disease progression and restitution has been found in cancer patients following SARS-CoV-2 Omicron variant infection. However, the interactions between the severity of COVID-19 in cancer patients with co-infection with Omicron variant and multiple clinical factors such as clinical features and systemic anticancer therapy have not been elucidated. Further in-depth studies on the relationship between antineoplastic therapy and COVID-19 severity are particularly limited and with controversial conclusions.

Unlike other earlier studies of COVID-19, this investigation was conducted in the context of the SARS-CoV-2 Omicron variant pandemic in China and focused on risk factors associated with the severity of COVID-19 caused by the Omicron variant. Our results showed that advanced age, male sex, poor physical status, and previous liver, kidney, and brain diseases were predictors of severe COVID-19. The factor of advanced age has been shown to be associated with an increased risk of death in several studies, but inconsistent findings exist for the sex factor (14, 15).

In the early stages of the COVID-19 pandemic, several studies (15–23) explored the impact of antineoplastic therapy on COVID-19, but the findings were inconsistent. In a multicenter retrospective study from Hubei Province of China (16), chemotherapy received within four weeks before the onset of COVID-19 symptoms was found to be a risk factor for death during hospital admission. Another large cohort study found (17) that certain chemotherapy regimens (e.g., R-CHOP, platinum combined with etoposide and DNA methyltransferase inhibitors) were associated with high 30-day all–cause mortality. Moreover, in a recently published retrospective cohort study (15), systemic anticancer therapy, particularly immunotherapy, was found to be associated with severe clinical outcomes in patients with cancer and COVID-19. However, a study from New York (18) showed that aggressive chemotherapy and radiotherapy were not associated with increased COVID-19 morbidity and mortality. Additionally, a study under the UK Coronavirus Cancer Surveillance Project (11) found no significant differences in the effects of chemotherapy within four weeks prior to diagnosis of new coronavirus pneumonia or of treatment modalities, such as immunotherapy, targeted therapy, or radiation therapy, on outcomes of more severe disease or death from COVID-19 pneumonia compared with cancer patients who did not receive chemotherapy during the same period. Other studies (14, 19) also indicated that recent anticancer therapy is not associated with adverse outcomes in COVID-19 infection.

Similarly, in the present study, we found no correlation between recent anticancer treatment status and the severity of COVID-19. The exact reason for these contradictory results is unclear, and it is speculated that these previous studies are from 2020 and earlier, when the prevalent viral strains were more pathogenic, which could be one of the reasons for finding that antineoplastic therapy can lead to critical illness. Another reason is that the patients included in different observations were very different. In some previous studies, the subjects contained a high percentage of hematologic tumors, and these patients may have been more susceptible to critical illness because of the poorer immunity of the organism due to the tumor and the intensity of treatment. However, a previous study specifically on patients with hematologic malignancies combined with COVID-19 showed (20) that recent cancer treatment did not significantly increase the risk of death.

In addition to analyzing the impact of anti-cancer modalities on the severity of COVID-19, we considered the possibility that anti-cancer treatment received within two weeks would worsen the immune function of cancer patients. Thus, we further analyzed the severity of severe disease in patients who had received anti-cancer treatment within two weeks compared with patients who had received treatment earlier. The first two weeks after the initiation of chemotherapy is a period of high incidence of chemotherapy-related adverse reactions and patients may experience severe bone marrow compromise, nausea and vomiting, etc. Curiously, in our study, we did not find that patients who received anti-tumor treatment, especially chemotherapy, within two weeks were more likely to develop more severe COVID-19.

There are several limitations to our study. First, it was retrospective. Despite the exhaustive follow-up examinations of the patients, the nature of the study may still lead to issues such as biased findings and missing data. Second, our study cohort included case data from two centers in China, Tangshan People’s Hospital and Yantai Yuhuangding Hospital. Although the overall sample size was large, the number of severe COVID-19 patients was small, which forced us to use univariate analysis for statistical analysis, resulting in a potential influence of confounding factors on the study results, affecting their reliability. Finally, we did not perform further sequencing of the patient’s strain to complete molecular typing. On the basis of the information provided by the government, we concluded that our patient had the Omicron variant. This also affected the rigor of the study. In the future, we will take measures to solve the above problems and improve the follow-up research.

## Conclusion

In conclusion, we identified several clinical factors that put cancer patients at high risk for severe COVID-19 infection, including advanced age, male sex, poor physical status, and previous liver, kidney, and brain diseases. However, the recent anticancer treatment profile, even if it had been implemented within two weeks, did not exacerbate the severity of COVID-19 caused by the Omicron variant. Therefore, cancer patients do not need to worry about severe disease caused by SARS-CoV-2 Omicron variant infection, while doctors should not interrupt anti-tumor treatment due to such concerns, especially targeted therapy.

## Data availability statement

The raw data supporting the conclusions of this article will be made available by the authors, without undue reservation.

## Ethics statement

The studies involving humans were approved by Ethics committee of Tangshan People’s Hospital. The studies were conducted in accordance with the local legislation and institutional requirements. The participants provided their written informed consent to participate in this study.

## Author contributions

YL: Writing – original draft, Writing – review & editing. WZ: Investigation, Writing – review & editing. ZW: Writing – original draft, Writing – review & editing. JY: Investigation, Writing – original draft. LD: Investigation, Writing – original draft. CL: Investigation, Writing – original draft. WW: Writing – original draft. FZ: Writing – original draft. SD: Writing – original draft. LS: Writing – original draft. ZY: Writing – original draft. CZ: Writing – original draft. FQ: Writing – original draft.
