# Supplementary material for: Recent antitumor therapy does not increase Omicron COVID-19 severity in cancer patients: a two-center retrospective study in China
Source: Front Oncol. 2023 Nov 21;13:1284255. doi: 10.3389/fonc.2023.1284255 (PMC10699754; doi:10.3389/fonc.2023.1284255)
Supplement: Supplementary file 1 [file Presentation_1.pdf]

Clinical Classification of Novel Coronavirus Infection (Trial Version 10) in China (Adult)

(I) Light.

Upper respiratory tract infection as the main manifestation, such as dry throat, sore throat, cough, fever, etc.

(ii) Medium-sized.

Persistent high fever > 3 days or (and) cough, shortness of breath, etc., but respiratory rate (RR) < 30 times / min, finger oxygen saturation > 93% when inhaling air at rest. Characteristic pneumonia manifestations of neocoronavirus infection are seen on imaging.

(iii) Heavy.

Adults with any of the following that cannot be explained by a cause other than neocoronavirus infection:

1. shortness of breath with RR >30 beats/min.

2. oxygen saturation <93% on air inhalation at rest.

3. Arterial partial pressure of oxygen (Pa02)/inhaled oxygen concentration (Fi02) <300mmHg (1mmHg=0.133kPa), high altitude (over 1000m above sea level) areas should be corrected for Pa0,/Fi0, according to the following formula: Pa0,/Fi0, x [760/atmosphere pressure (mmHg)].

4. Progressive worsening of clinical symptoms and lung imaging showing significant progression of the lesion >50% within 24-48 hours.

(iv) Critically ill type.

Those who meet one of the following conditions:

1. the presence of respiratory failure and the need for mechanical ventilation;

2. the presence of shock;

3. Combined with other organ failure requiring ICU monitoring treatment.
